# Military sexual trauma and lower relationship satisfaction are associated with suicide risk among male service members and veterans

**DOI:** 10.3389/fpsyt.2024.1355355

**Published:** 2024-05-31

**Authors:** Whitney S. Livingston, Rebecca K. Blais

**Affiliations:** ^1^ Department of Psychology, Utah State University, Logan, UT, United States; ^2^ Department of Psychology, Arizona State University, Tempe, AZ, United States

**Keywords:** sexual trauma, suicide risk, veterans, males, couple, service members, relationship satisfaction

## Abstract

Male service members/veterans die by suicide at increased rates relative to civilians and females in the military, with risk increasing following military sexual trauma (MST) exposure. Suicide theories emphasize the role of feeling connected to others, and in the context of romantic relationships, it is possible that higher relationship satisfaction buffers the effects of MST. That said, MST exposure is associated with higher relationship distress, so the potential buffering effects are unclear. The current brief report assessed the interaction of relationship satisfaction and MST exposure as correlates of suicide risk among a convenience sample of 290 partnered male service members/veterans. This secondary analysis utilized a survey to assess MST exposure, relationship satisfaction, suicide risk, and demographics. Using linear regression, suicide risk was regressed on MST exposure, relationship satisfaction, and their interaction, as well as demographic covariates. The average score for relationship satisfaction suggested distressed relationships (*M*=13.41, *SD*= 4.55) and 16.21% (*n=*47) reported MST. Suicide risk was elevated (*M*=5.95, *SD*=3.23). The linear regression revealed that MST exposure (*B*=1.21, *p*=.02) and lower relationship satisfaction (*B*=-0.97, *p*<.001) were individually associated with higher suicide risk; however, their interaction was non-significant (*p*>.05). MST exposure and satisfaction in one’s romantic relationship have unique and separate associations with suicide risk. Relationship satisfaction did not buffer the effects of MST on suicide, and this may be due to overall poor satisfaction scores. Notwithstanding, findings highlight the need to address both MST exposure and relationship satisfaction to reduce risk of suicide.

## 1 Introduction

Prevention of suicide is identified as a “top clinical priority” by the United States (U.S.) Department of Veterans Affairs (VA) (1), and among those in the U.S. military, suicide is one of the leading causes of preventable death (2). In 2021, 24.3 service members out of every 100,000 died by suicide (3). Males in the military are at particularly heightened risk for suicide, with 94% of suicide deaths accounted for by males in Active Duty (3). Higher risk for death by suicide among males continues after discharge, such that 33.7 male veterans relative to 13.8 female veterans died by suicide per 100,000 in 2020 (4). Research posits that increased use of lethal means such as firearms (5), reduced fear due to increased exposure to violence (e.g., through guns and violent sports) (6, 7), and increased pain tolerance (8) may account for some of this elevated risk.

Specific military factors also contribute to overall heightened risk. In particular, military sexual trauma (MST), which includes experiencing any uninvited or unwanted sexual harassment or assault during military service, is associated with higher risk for death by suicide (9). The DoD reported that of male service members, 5.7% experienced military sexual harassment and 0.6% experienced military sexual assault (10). The VA combines military sexual harassment and assault into MST, and reports that 1.6% of male veterans experienced MST (11). These estimates are low as evidence suggests there are reporting and disclosure barriers during the screening process (12, 13). Studies of suicide risk among male MST survivors are notably limited (14). Given their overall risk for death by suicide, additional studies in males are critically needed to better understand this phenomenon.

Several theories of suicide risk point to the importance of strong interpersonal connections in preventing suicide (15–17), and these theories may be particularly insightful for male veterans who are partnered and experienced MST. Previous research shows that relationship satisfaction is lower among those who report suicide risk, but findings are generally circumscribed to civilians (18), female service members/veterans (19), or those specifically in the National Guard (20). In the context of MST, service members/veterans who were married had higher suicide risk, suggesting that romantic relationships could be an important factor to consider (9). Whereas one might expect higher relationship satisfaction to buffer the effects of MST on suicide, the presence of MST is associated with disrupted romantic relationship function (21, 22). As such, any potentially buffering effects of relationship satisfaction in preventing suicide may be neutralized by MST exposure.

The current study adds to the literature on suicide risk by examining whether an interaction of MST exposure and relationship satisfaction is associated with heightened suicide risk in male service members/veterans. We hypothesized that males who reported MST exposure and lower relationship satisfaction would have the highest risk for suicide after accounting for demographic, military, and related trauma exposure factors.

## 2 Methods and measures

### 2.1 Participants

The current study extracted data from a parent study aimed at examining sexual function and MST among 508 male service members/veterans (23). Participants from the parent study were included in this current study if they reported being partnered or married at the time of participation, and if they completed measures on suicide risk, MST, relationship satisfaction, and included covariates. This resulted in a final sample of 290 partnered male service members/veterans.

### 2.2 Procedure

Participants were recruited through social media advertisements. Individuals interested in participating were directed to a Qualtrics survey, where they completed screening questions confirming they identified as male, were of consenting age (≥18), were currently in a partnered or married relationship, and were actively serving in the military or were veterans. Participants that met these inclusion criteria were provided with all study details through an Institutional Review Board Letter of Information (LoI). Those who wanted to continue with the study following the LoI provided consent. Participants were offered $15 compensation. This study was approved by the Utah State University Institutional Review Board.

### 2.3 Measures

Suicide risk was assessed with the *Suicide Behaviors Questionnaire-Revised* (SBQ-R) (24). The SBQ-R is a 4-item self-report measure that examines various aspects of suicide risk, including recent and lifetime suicide ideations and attempts, and perceived likelihood of future suicide. Participants indicated their response through Likert scales, which vary for each question. For instance, the second item states, “How often have you thought of killing yourself in the past year?” and provides Likert responses of 1 (*never)* to 5 (*very often, 5 or more times*). Responses are summed to create total scores, which range from 3 to 18, with higher scores indicating increased risk of suicide. Cronbach’s alpha in the current sample was adequate, .82.

MST was assessed through the VA Military Sexual Trauma Screening Questionnaire. This is a two-item self-report screener that identifies unwanted/uninvited sexual attention, harassment, and/or assault that occurs during military service. The two items are, “When you were in the military, did you receive uninvited and unwanted sexual attention?” and “Did someone ever use force or threat of force to have sexual contact with you against your will?” Affirmative responses to one or both of these items were coded as 1 (MST), whereas participants who denied having these experiences were scored as 0 (no MST).

Relationship satisfaction was assessed through the *Couples Satisfaction Index-4* (CSI-4) (25). The CSI-4 is a four-item self-report measure that assesses relationship satisfaction. A sample item includes “In general, how satisfied are you with your relationship?” The four items were scored on a Likert scale with varying anchors that range from 0-6 or 0-5. The total score is calculated by summing the four responses. The total scores range from 0 to 21, with higher scores indicating greater levels of satisfaction in the relationship. The CSI-4 is a valid and reliable measure (25). Cronbach’s alpha for the CSI-4 using data from the current sample was adequate, .87.

For the current study, age, race and ethnicity (African American/Black, American Indian/Alaska Native, Latino/Hispanic, Bi-racial/Multi-racial, and White), military branch (Marines, Navy, Air Force, Coast Guard, and Army), and premilitary sexual trauma were included as covariates and were assessed through a demographic inventory included in the parent study. Marital status (married and non-married) and discharge status (active duty and veteran status) are reported to describe the composition of the sample. While the authors intended to examine the increased risk of suicide for each race/ethnicity and military branch, the number of participants who endorsed various races/ethnicities and military branches were too small to examine. Thus, the race/ethnicity variable was coded as 1 (minority race/ethnicity) and 0 (White race) as the majority of participants identified as White. Additionally, military branch was coded as 1 (Army) and 0 (all other branches) as the majority of participants identified as serving in the Army branch. Exposure to premilitary sexual trauma was dummy coded 1 (premilitary sexual trauma) and 0 (no premilitary sexual trauma).

### 2.4 Data analysis

**Figure 1:**
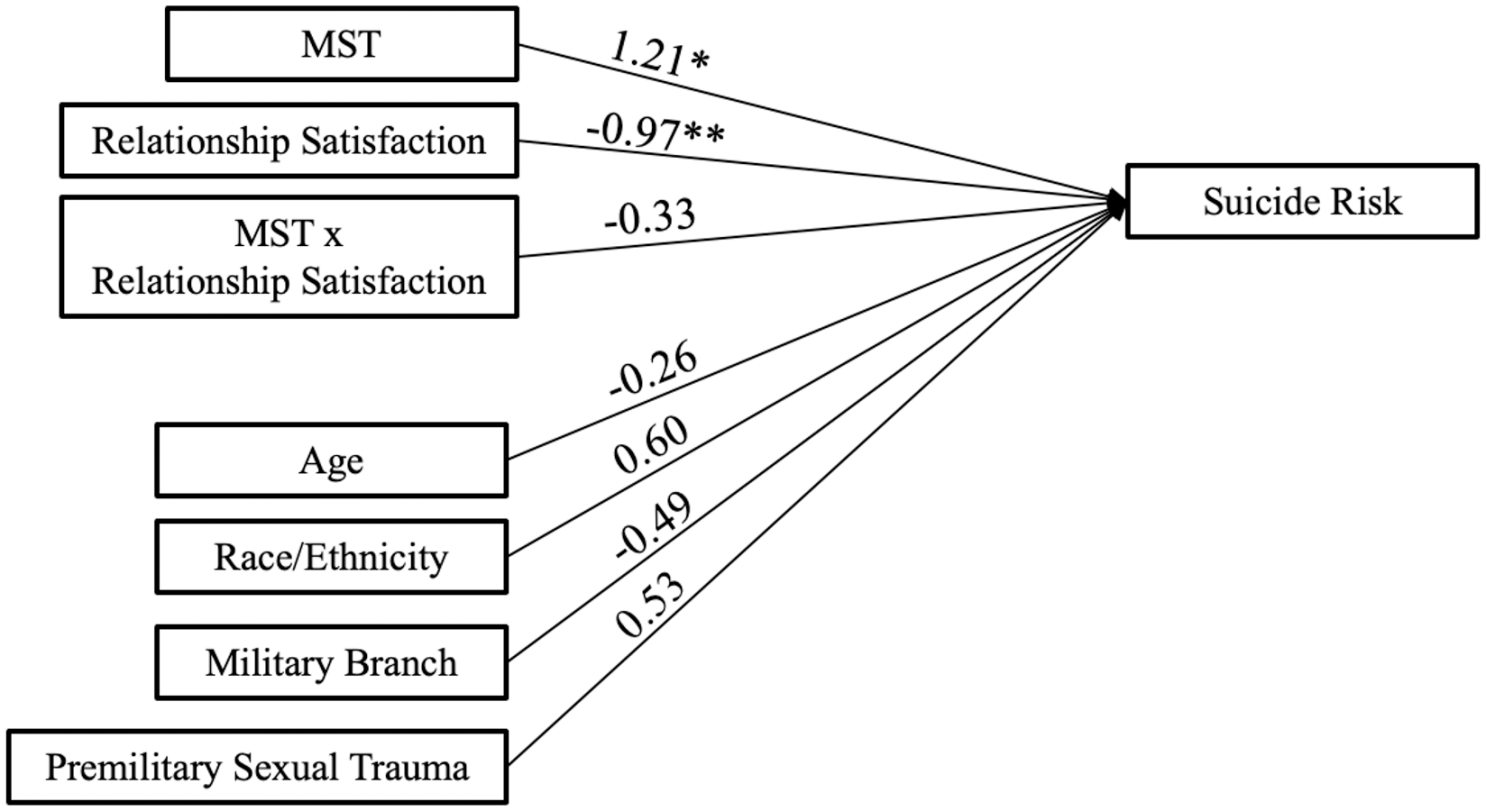
Military sexual trauma = MST. *p<.05, **p<.001.

Sample characteristics were examined using descriptive statistics. Pearson correlations tested differences between continuous variables. *T-*tests were used to examine differences in the continuous variables of suicide risk and connectedness across those with and without history of MST. Linear regression was used to test the associations shown in **Figure 1**. Continuous exogenous variables of relationship satisfaction and age were centered and scaled. Direct paths were calculated from exogenous variables of MST, relationship satisfaction, and their interaction to suicide risk. Direct paths from covariates to suicide risk were included. Data were analyzed through the R environment (26).

## 3 Results

### 3.1 Sample characteristics

The majority of the sample identified as White (*n*=257, 88.62%), with other participants identifying as African American/Black (*n*=12, 4.14%), Native American/Alaskan Native (*n*=9, 3.10%), Latino/Hispanic (*n*=16, 5.52%), bi-racial/multi-racial (*n*=11, 3.79%) or selecting “other” race/ethnicity (*n*=2, 0.70%). The majority reported service in the Army (*n*=180, 62.07%), with other participants reporting service in the Air Force (*n*=51, 17.59%), Navy (*n*=53, 18.28%), Marines (*n*=21, 7.24%), or multiple branches (*n*=15, 5.17%). Participants could indicate more than one racial and/or ethnic identity and more than one service branch, thus, the sums of each are higher than the number of service members/veterans included in the study. The average age was 38.79 (standard deviation [*SD*]=11.00), and the majority of the sample were married (*n*=220, 75.86%), and veterans (*n*=218, 75.17%). Men service members/veterans on average scored 5.95 (*SD*=3.23) on the measure assessing suicide risk (SBQ-R) and 13.41 (*SD*=4.55) on the measure assessing relationship satisfaction (CSI-4). Of the men service members/veterans who responded to the SBQ-R, 40.00% (*n=*116) reported suicide ideation in the past year. A minority of participants reported experiencing MST (*n=*47, 16.21%), premilitary sexual trauma (*n*=52, 17.93%), and both MST and premilitary sexual trauma (*n*=22, 7.59%).

### 3.2 Bivariate associations

Bivariate analyses revealed higher suicide risk was significantly associated with lower relationship satisfaction with a moderate effect size *(r*=-0.33, *p*<.001). Results of independent sample *t*-tests between MST exposure and continuous study variables indicated suicide risk was higher among those who reported MST (*M*=7.45, SD=3.89) relative to those who did not report MST (*M*=5.66, *SD*=3.01; *t*=-2.98, *p*=.004). There was no significant difference in relationship satisfaction between those with (*M*=12.43, *SD*=5.18) and without experiences of MST (*M*=13.60, *SD*=4.41; *t*=1.62, *p*=.11). There was no significant difference in relationship satisfaction between partnered participants (*M*=13.00, *SD*=4.39) and married participants (*M*=13.54, *SD*=4.60; *t*=-0.86, *p*=.391). Moreover, rates of MST were not different between those who were partnered (*n* = 14, 20.00%) versus married (*n* = 33, 15.00%; *p* = .64).

### 3.3 Regression analysis

The regression model and estimates depicted in **Figure 1** indicated MST exposure was associated with higher suicide risk, as was lower relationship satisfaction; however, the interaction of MST and relationship satisfaction was not related to suicide risk. Covariates were not related to suicide risk.

## 4 Discussion

The results of this study explored whether a combination of MST exposure and relationship satisfaction were associated with higher suicide risk among male service members/veterans. Contrary to hypotheses, the presence of both lower relationship satisfaction and MST exposure did not increase risk for suicide, yet both poor relationship satisfaction and MST exposure were independently associated with higher suicide risk in partnered males. The association of lower relationship satisfaction with suicide risk (19, 20) and MST exposure with suicide risk (9) is consistent with previous literature. It is possible that relationship satisfaction did not buffer the effects of MST on suicide risk in the current sample due to a low average level of satisfaction reported by the participants.

Many suicide interventions are focused on the individual, despite theories identifying a lack of connectedness or perceived burden to others as a core predictor of suicide (15–17). Previous research considered increasing peer support to reduce suicide risk among veterans (27–29), and our findings suggest it may be critical to incorporate more couples therapy interventions. Research suggests that the majority of veterans who previously talked to their partner about their suicidal thoughts would be interested in a couples-based suicide intervention (30), and preliminary findings from a couples-based suicide intervention show improved feelings of belonging and reduced suicide risk (31). These findings show good promise for the utility of including partners in these efforts.

There are limitations to the current study. The data were extracted from a convenience sample of partnered male service members/veterans. This suggests that findings from this study may not be generalizable to female or single male service members/veterans. Moreover, the majority of participants identified as White race (*n*=257, 88.62%), which is similar to the composition of men in the military; however, future research would benefit from oversampling of individuals from racially and ethnically marginalized communities to better understand whether results are consistent and generalizable. Data collection was cross-sectional, therefore, temporality cannot be assumed. The SBQ-R scores are skewed right and not normally distributed (Shapiro-Wilk test, *W*=0.85, *p*<.001). This may increase the difficulty of identifying a significant interactive effect as hypothesized. The proportion of participants who experienced both MST and premilitary sexual trauma was small (7.59%), however, future research with larger samples may explore how sexual revictimization increases risk of suicide.

The current study contributes to the literature factors that are related to suicide risk among males. In particular, we observed that MST exposure and lower relationship satisfaction were each associated with higher suicide risk but their combination did not further attenuate this public health concern. This study suggests that improving connectedness to one’s partner among men service members/veterans may reduce suicide risk, and underscores the critical need to focus on the sequelae of MST.

## Data availability statement

The original contributions presented in the study are included in the article/Supplementary Materials. Further inquiries can be directed to the corresponding author.

## Ethics statement

The studies involving humans were approved by Utah State University Institutional Review Board. The studies were conducted in accordance with the local legislation and institutional requirements. The participants provided their written informed consent to participate in this study.

## Author contributions

WL: Writing – original draft, Methodology, Formal analysis, Conceptualization. RB: Writing – review & editing, Supervision, Project administration, Methodology, Investigation, Funding acquisition, Conceptualization.
